# Brain augmentation and neuroscience technologies: current applications, challenges, ethics and future prospects

**DOI:** 10.3389/fnsys.2022.1000495

**Published:** 2022-09-23

**Authors:** Nitish Singh Jangwan, Ghulam Md Ashraf, Veerma Ram, Vinod Singh, Badrah S. Alghamdi, Adel Mohammad Abuzenadah, Mamta F. Singh

**Affiliations:** ^1^Department of Pharmacology, School of Pharmaceutical Sciences and Technology, Sardar Bhagwan Singh University, Balawala, India; ^2^Pre-Clinical Research Unit, King Fahd Medical Research Center, King Abdulaziz University, Jeddah, Saudi Arabia; ^3^Department of Medical Laboratory Sciences, Faculty of Applied Medical Sciences, King Abdulaziz University, Jeddah, Saudi Arabia; ^4^Prabha Harji Lal College of Pharmacy and Paraclinical Sciences, University of Jammu, Jammu, India; ^5^Department of Physiology, Neuroscience Unit, Faculty of Medicine, King Abdulaziz University, Jeddah, Saudi Arabia; ^6^Faculty of Applied Medical Sciences, King Abdulaziz University, Jeddah, Saudi Arabia; ^7^King Fahd Medical Research Center, King Abdulaziz University, Jeddah, Saudi Arabia

**Keywords:** brain 2025, brain machine interface, deep brain stimulation, ethics, non-invasive and invasive brain stimulation

## Abstract

Ever since the dawn of antiquity, people have strived to improve their cognitive abilities. From the advent of the wheel to the development of artificial intelligence, technology has had a profound leverage on civilization. Cognitive enhancement or augmentation of brain functions has become a trending topic both in academic and public debates in improving physical and mental abilities. The last years have seen a plethora of suggestions for boosting cognitive functions and biochemical, physical, and behavioral strategies are being explored in the field of cognitive enhancement. Despite expansion of behavioral and biochemical approaches, various physical strategies are known to boost mental abilities in diseased and healthy individuals. Clinical applications of neuroscience technologies offer alternatives to pharmaceutical approaches and devices for diseases that have been fatal, so far. Importantly, the distinctive aspect of these technologies, which shapes their existing and anticipated participation in brain augmentations, is used to compare and contrast them. As a preview of the next two decades of progress in brain augmentation, this article presents a plausible estimation of the many neuroscience technologies, their virtues, demerits, and applications. The review also focuses on the ethical implications and challenges linked to modern neuroscientific technology. There are times when it looks as if ethics discussions are more concerned with the hypothetical than with the factual. We conclude by providing recommendations for potential future studies and development areas, taking into account future advancements in neuroscience innovation for brain enhancement, analyzing historical patterns, considering neuroethics and looking at other related forecasts.

## Introduction

Humans have striven to increase their mental capacities since ancient times. From symbolic language, writing and the printing press to mathematics, calculators and computers, mankind has devised and employed tools to record, store, and exchange thoughts and to enhance cognition. Revolutionary changes are occurring in the health care delivery system as a result of the accelerating speed of innovation and increased employment of technology to suit society’s evolving health care needs (Sullivan and Hagen, 2002). The aim of researchers working on cognitive enhancement is to understand the neurobiological and psychological mechanisms underlying cognitive capacities while theorists are rather interested in their social and ethical implications (Dresler et al., 2019; Oxley et al., 2021). “Augmentation of brain function,” is an umbrella term for the approaches from different disciplines, aimed at the improvement of brain performance in both healthy people and patients suffering from neurological disabilities. Brain augmentation was first reported in 1874 in humans. In 1924, Hanns Berger invented Electroencephalography (EEG), a significant advancement for humans that enabled researchers to record human brain activity. Later on with advancements in technology, various brain computer interface (BCI) techniques like Cyborg Insects, Cyborg Sharks, Cyberkinetics, NeuroPort^TM^, Brain Gate, Neuralink, and Neural Lace implant were introduced with the goal of developing an ultra-high bandwidth brain-machine interface. Augmentative technologies can improve both physical and mental abilities. Depending upon the extent of invasiveness, various neuroscience technologies are available that have potential for tracking and altering brain activities. Clinical applications of neurotechnologies offer alternatives to pharmaceutical approaches and devices for diseases that have been fatal, so far. Brain augmentation techniques, approaches, and technologies can also boost human abilities in those without any disease. Augmentation techniques like Transcranial magnetic stimulation (TMS) was originally used to investigate and diagnose neurological injury but recently the applications of TMS in otherwise “healthy” people are expanding and include boosting attention and vigilance, motor learning, improving attention, cognition and many more (Moss and Scholey, 1996; Glade, 2010; Dresler et al., 2013; Yu et al., 2015). Deep brain stimulation (DBS), TMS, and focused ultrasound (FUS) are few neuroscience technologies that are gaining attention at present. As per transhumanist literature, technological augmentation of “normal” human function moves us away from our species functional limitations and closer to “super” human function (Smith et al., 2011). Future applications of emerging technology can transform us into *Homo sapiens technologicus*—a species that uses, fuses, and integrates technology to enhance its own function. However, as technology and society are always intertwined, the risk factors associated with the brain augmentation cannot be overlooked. Just like there is a culture of hacking when it comes to computer software, more and more people are experimenting with ways to get around the natural limits of human cognitive capacity called “hacking brain function”. This development has led to both enthusiasm and dread, as socialists and scientist have different opinion about the feasibility, utility, risks, and eventual impact of enhancement technologies on the world (Dresler et al., 2019). Finally, with every new step in the development of technologies, there will be a potential for abuses. A variety of science fiction scenarios involving cyborgs and the imminent transformation of the human race into a semi-electronic species has left the public confused against scientific progress (Short, 2005). Only with a clear image of how a certain enhancement method might alter cognitive processes in specific populations, along with side effects and costs, can make it justifiable.

In this review article, authors discuss physical strategies enhancement approaches of brain augmentation technique as one of the subset including their applications in neurological diseases as well as non-medical applications. The authors take look at the most common neuroscientific methods for monitoring and manipulating brain activity, which are essential for human cognitive enhancement. Purpose of this review is to draw attention about the uses of this emerging revolutionary technology, its challenges, limitations including ethical issues associated with these techniques, and future states of brain augmentation techniques.

## Chronological Development in The Field of Brain Augmentation

**Figure 1:**
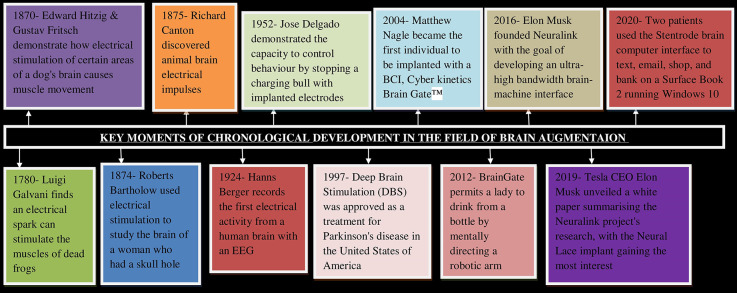
Milestones of chronological development in the field of Brain Augmentation. Key moments in the history of brain computer interface which eventually leads to the development of brain augmentation techniques for the welfare of humanity so as to mitigate several diseases and neurological disorders. BCI, Brain computer interface; EEG, Electroencephalogram; DBS, Deep brain stimulation.

Figure 1 depicts different stages of chronological developments in the field of brain augmentation. The diagram depicts the evolution of brain augmentation and neuroscience techniques from their origins to their widespread applications in the modern technological world. Brain augmentation is an ancient technique as in 1780, Luigi Galvani found that an electrical spark can stimulate the muscles of dead frog. Brain augmentation in human was reported in 1874 when Roberts Bartholow conducted a study on the brain of a woman who had a hole in her head using electrical stimulation (Patra et al., 2019). In 1924, Hanns Berger recorded the first electrical activity in a human brain with an EEG and later Berger was credited with inventing electroencephalography, a significant advancement for humans that enabled researchers to record human brain activity (Millett, 2001).

In 2000, US military projects created implants in the brains of animals *via* Micro-Electro-Mechanical Systems that allowed for effective animal control. Such study resulted in the emergence of “Cyborg Insects” and “Cyborg Sharks” (Meera and Neethu, 2015). In 2001, John Donoghue and a group of Brown University researchers founded Cyberkinetics, a public traded corporation to commercialize a brain-computer interface. NeuroPort^TM^ is the company’s first commercial product. With the help of NeuroPort^TM^ Neural Monitoring System researchers were able to detect microseizure activity in patients prior to epileptic seizures (*The History of Brain Machine Implants*, n.d.). In 2012, Brain Gate was introduced (Orenstein, 2021; Wikipedia contributors, 2022). In 2016, Elon Musk founded Neuralink with the goal of developing an ultra-high bandwidth brain-machine interface and in 2019 Neural Lace implant was introduced. The implant would theoretically grow inside the brain alongside the brain, forming an artificial intelligence layer on top of the brain to augment its activities (Musk and Neuralink, 2019). In October 2020, two patients were able to use the Stentrode brain computer interface to remotely control a Surface Book 2 running Windows 10 to text, email, shop, and bank. This was the first time a brain–computer interface was implanted through the patient’s blood vessels, so avoiding open-brain surgery (Oxley et al., 2021).

## Cognitive Enhancement Approaches

**Figure 2:**
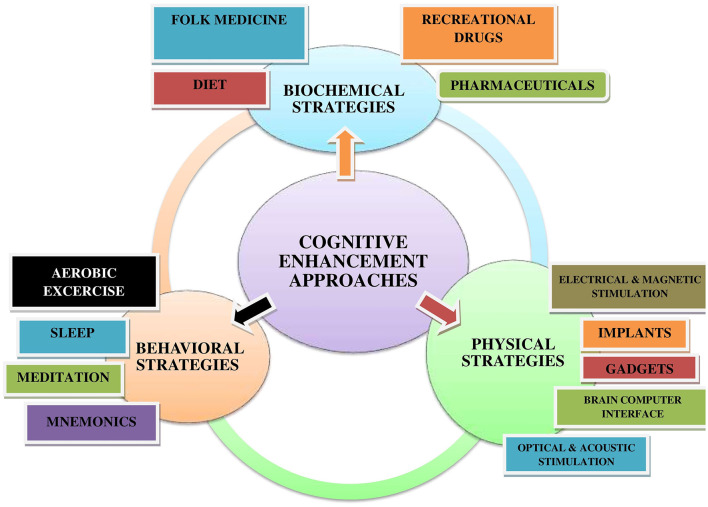
Diagrammatic illustration of different cognitive enhancement approaches. As per the mode of action, biochemical, behavioral, and physical strategies are generally used to enhance cognition and brain augmentation.

Enhancement is described as human interventions that try to improve mental functioning above and beyond what is required to maintain or restore health. The non-pharmacological techniques to cognitive enhancement solutions are classified into three broad categories based on their primary mode of action (Dresler et al., 2013, 2019). Figure 2 demonstrates that, the majority of cognitive enhancement approaches can be classified as biochemical, physical, or behavioral interventions. Biochemical approaches include the use of either traditional medicines or pharmaceuticals. Behavioral approaches include lifestyle modifications, whereas physical approaches include non-invasive and invasive brain stimulation techniques.

### Biochemical strategies

Biochemical interventions are not limited to “smart medications” in the pharmaceutical sector (Moss and Scholey, 1996; Yu et al., 2015). Biochemical enhancers have a long history in human history as ways for using specific food components. The most often utilized substances are undoubtedly glucose and caffeine, both of which have been shown in multiple trials to enhance cognition (Glade, 2010; Smith et al., 2011). Additionally, flavonoids have been shown to have cognitive boosting benefits (Rendeiro et al., 2012; Socci et al., 2017). Apart from specialized dietary supplements, fasting and general calorie restriction have been shown to improve memory in elderly individuals (Dresler et al., 2019). Certain traditional natural therapies have also been identified as cognitive enhancers, most notably traditional Chinese and Indian herbal medicines such as *Bacopa monnieri* (Howes and Houghton, 2003; Kongkeaw et al., 2014). Another long-established biochemical intervention is the usage of drugs for recreational purposes that have been shown to increase specific cognitive functions (Warburton, 1992; Benedek et al., 2017). Cosmetic neurology is another approach that uses brain enhancement with the help of chemicals like nootropic drugs (Nishizaki et al., 1999; Dees, 2004). Nootropics can be natural or synthetic that improves concentration, memory, and cognitive function by stimulating and targeting neurochemistry (Turner et al., 2003; Dielenberg, 2013). Pharmaceuticals such as amphetamine, methylphenidate, or modafinil, as well as antidementia medications such as acetylcholinesterase inhibitors and memantine, are at the center of public debate about cognitive enhancement. However, the data supporting their efficiency in enhancing brain function and cognition in healthy persons is frequently far less than what is predicted in theoretical debates (Repantis et al., 2010a, b; Fond et al., 2015). One more drawback with the use of nootropics is that, human brain is probably not adapted to excessive pharmaceutical medicated modification of brain neurochemistry (Sullivan and Hagen, 2002). Additional pharmacological strategies for cognitive enhancement include genetic changes, which have been shown to boost a variety of learning and memory activities in animal models (Tang et al., 1999).

### Behavioral strategies

Although not often acknowledged as such by the general public, the most widely used and longest-lasting cognitive enhancers are almost certainly behavioral strategies. An increasing body of evidence demonstrates that routine activities such as sleep and physical activities boost cognitive performance (Hötting and Röder, 2013; Diekelmann, 2014). Additionally, well-established cultural activities like musical training, dance, or learning a second language have been shown to boost cognition in ways that are not directly related to the abilities being practiced (Bialystok et al., 2012; Seinfeld et al., 2013). Along with these natural and culturally accepted activities, numerous behavioral techniques have been devised to actively increase certain brain processes. Mnemonic approaches for improving learning and memory, as well as meditation training for improving attention processes and mindfulness, are two methodologies that date all the way back to ancient times (Worthen and Hunt, 2010; Sedlmeier et al., 2012). Commercial video games and personalized computer training are relatively recent innovations aimed at enhancing certain cognitive capacities and skills (Green and Bavelier, 2012; Lampit et al., 2014).

### Physical strategies

Brain stimulation techniques are currently the most commonly discussed physical strategies for cognitive enhancement. While invasive methods such as DBS have been shown to improve cognition in subjects with pathological conditions, several allegedly noninvasive stimulation strategies, including electrical stimulation methods such as transcranial direct current stimulation (tDCS), transcranial alternating current stimulation (tACS), transcranial random noise stimulation (tRNS), and transcranial pulsed current stimulation (tPCS), transcutaneous vagus nerve stimulation (tVNS), or median nerve stimulation (MNS) are increasingly used on healthy subjects (Cinel et al., 2019; Ke et al., 2019). Apart from electrical stimulation methods, a potential for cognitive enhancement has been reported for TMS, optical stimulation with lasers, and several forms of acoustic stimulations, including transcranial focused ultrasound stimulation, binaural beats, or auditory stimulation of the EEG theta rhythm or sleep EEG slow oscillations (Gonzalez-Lima and Barrett, 2014; Luber and Lisanby, 2014). Physical enhancement techniques that indirectly target brain functions include whole-body vibrations, stochastic resonance motor control improvement, and various forms of neurofeedback, such as EEG neurofeedback in the upper alpha band for memory, working memory, and visuospatial abilities augmentation. Recent research has demonstrated that the use of Functional magnetic resonance imaging (fMRI) neurofeedback combined with multivariate pattern analysis has the potential to improve sustained attention or visuospatial memory (Cinel et al., 2019). However, all of these techniques have their advantages and disadvantages. Table 1 discusses the benefits and drawbacks of numerous neuroscience techniques for monitoring brain activities. Neural implants or prosthesis for the brain have advanced in controlled laboratory conditions that may aid in human memory (Warwick, 2018).

**Table 1:** Pros and Cons of various neuroscientific tehcniques for monitoring and altering brain activities.

**DisorderTechnology**	**Nature of technology**	**Pros**	**Cons**
**EEG (Recording technology)**	NON INVASIVE	» Economical » Handy » Incredible temporal resolution	» Restricted spatial resolution » Only measures neural activity near the scalp
**MEG (Recording technology)**	NON INVASIVE	» Good temporal resolution » Contactless (with the body)	» Costly » Colossal and immobile
**fNIRS (Recording technology)**	NON INVASIVE	» Economical » Handy	» Laborious calibration » Low spatial and temporal resolution
**fMRI (Recording technology)**	NON INVASIVE	» Good spatial resolution » No physical contact with body	» High price » Poor temporal resolution » Colossal and immobile
**ECoG (Recording technology)**	INVASIVE	» Fine signal quality » Satisfactory temporal and spatial resolution	» High cost » Neurosurgery required
**Implanted micro-electrodes (Recording and stimulation technology)**	INVASIVE	» Fine signal quality » High definition temporal and spatial resolution	» Narrow coverage of brain area » Neurosurgery required
**DBS (Stimulation technology)**	INVASIVE	» High definition temporal and spatial resolution » stimulation of extensive brain regions permitted	» Threat associated with surgery (e.g., infections, interaction with brain neurons) » Neuropsychiatric side effects
**tES (Stimulation technology)**	NON INVASIVE	» Economical » Handy » Good spatial resolution for high-magnification tES	» Poor spatial resolution for normal tES » Long-term repercussions are still a mystery
**TMS (Stimulation technology)**	NON INVASIVE	» High degree of spatial and temporal resolution	» Costly » Bulky and immobile
**FUS (Stimulation technology)**	NON INVASIVE	» High degree of spatial and temporal resolution	» Inadequate clinical trials » Applicable to limited part of brain

BCI, a physical strategy for cognitive improvement that connects neural circuits to external support devices, is the most prevalent method. By decoding neural recordings and transmitting sensory signals to the brain, BCIs may communicate with external devices and provide commands for them (Saha et al., 2021). The emerging field of BCI technology may allow individuals unable to speak and/or use their limbs to once again communicate or operate assistive devices for walking and manipulating objects (McFarland and Wolpaw, 2011; Nicolas-Alonso and Gomez-Gil, 2012).

## Neuroscience Technologies for Recording and Influencing Brain Activities

The ability to record and stimulate brain activity has revolutionized our understanding of cognitive mechanisms related to perception, memory, attention, action planning, and execution. However, whether or not these approaches can be used for cognitive enhancement depends not only on their ability to detect and/or stimulate specific brain areas, but also on a variety of other factors. The degree of invasiveness, that is how much a technology involves insertion of equipment into the body as well as other practical aspects such as portability and cost, has impact on usability of the technology for ordinary human cognitive enhancement (Müller and Rotter, 2017; Cinel et al., 2019; Goering et al., 2021).

### Neuroscience technologies for recording

#### Non-invasive recording technologies

EEG, fMRI, fNIRS, and MEG are the most widely used non-invasive techniques for recording brain activity (Cinel et al., 2019). EEG records electrical activity *via* electrodes positioned on the head. One of the primary advantage of EEG is its high temporal resolution, low cost, portability, and ease of use, all of which are critical when considering its usability outside the lab for cognitive enhancement. The spatial resolution, on the other hand, is typically low (Sheehy, 1984; Schomer and Da Silva, 2012).

fMRI detects variations in the blood flow (hemodynamic response) in the brain to determine brain activity. It has far higher resolution than EEG, but low temporal resolution. Regrettably, fMRI requires large, expensive equipment to acquire signals and is inappropriate for human brain enhancement (Weiskopf et al., 2004; Luck et al., 2019).

fNIRS, like fMRI, measures the location and intensity of brain activity *via* hemodynamic responses. Its primary advantages is it’s portability and is less vulnerable to electrical noise than fMRI and EEG (Irani et al., 2007). Due to these factors fNIRS is quite useful for cognitive enhancement applications in humans, particularly when combined with brain stimulation technologies, such as those used to improve spatial working memory. fNIRS, on the other hand, has a limited spatial and temporal resolution (Ferrari and Quaresima, 2012; McKendrick et al., 2015).

Another non-invasive technique is MEG, which is often used to assess the function of various brain regions, locate areas impacted by pathology, and for other medical purposes. MEG, like fMRI, requires a magnetically insulated laboratory, and is costly (Hämäläinen et al., 1993; Ahn et al., 2013).

#### Invasive recording technologies

In invasive techniques, electrodes are put directly into or on the surface of the brain. As a result, the recordings are less impacted by noise and distortions caused by the scalp and skull, and have a high temporal and spatial resolution. However, implanting electrodes in brain requires brain surgery, which increases the cost of these procedures and raises significant ethical concerns (Cinel et al., 2019). Electrocorticography (ECoG) is one of the invasive technology. It is similar to EEG in that it uses electrodes to monitor the electrical activity generated by neurons, but unlike EEG, the electrodes are put directly in the cortex. Additionally, ECoG typically detects neuronal activity from a very limited region of the cortex. Nonetheless, applications for human cognitive enhancement based on ECoG exist (Wyler, 1987).

Insertion of arrays of needle-shaped microelectrodes in the brain is another invasive recording technique. It provides high-quality signals that are just moderately influenced by noise and are extremely detailed (i.e., each electrode measures the electrical activity of one or very few neurons; Oka et al., 1999). Among the invasive electrodes, Gerhardt and associates’ ceramic-based microelectrodes are the most widely used electrodes in brain recording (Burmeister et al., 2000). Due to their elongated shape and the existence of numerous pads on their surface, the electrodes enable high-precision and high-density multi-recordings in deep brain areas, as well as electrical stimulation (Hampson et al., 2013; Opris et al., 2015). Major disadvantage of invasive recording methods was that they typically cover only a small portion of the brain; however, recent developments have enabled examination of considerably larger regions. Due to the hazards and ethical concerns with neurosurgery, majority of research involving microelectrodes has been conducted on non-human primates or rodents. Human research has been limited to individuals with motor difficulties (Borton et al., 2013; Waldert, 2016; Pesaran et al., 2018).

### Brain stimulation technologies

Brain stimulation technologies are established neurosurgical techniques that stimulate brain tissues and used to treat several neurological disorders, including Parkinson’s disease, pain, movement disorders like tremor and dystonia as well as epilepsy and psychiatric disorders (Caulfield and George, 2018).

#### Non-invasive stimulation technologies

Transcranial electrical stimulation (tES), transcranial magnetic stimulation (TMS), and focused ultrasound (FUS) are the most often used non-invasive brain stimulation modalities (Cinel et al., 2019). Stimulating brain with tES includes connecting electrodes to the scalp and injecting a modest direct (transcranial Direct Current Stimulation, tDCS) or alternating (transcranial Alternating Current Stimulation, tACS) current (1–2 mA in strength) for up to 30 min. As compared to other techniques, tES is more affordable and portable. However, it has the restriction of a low spatial resolution. While tES has demonstrated promising outcomes in human brain enhancement, concerns have been expressed regarding its true non-invasiveness, the implications of continuous use, and the variability of result outcomes among various subjects (Moreno-Duarte et al., 2014; Reed and Cohen Kadosh, 2018).

TMS creates a magnetic field around a coil positioned on the participant’s scalp and facilitates flow of current in the underlying cortical tissue, thereby modifying neuronal activity. However, all contemporary TMS designs have significant limitations (Pascual-Leone et al., 1995; Epstein, 2014). First, coils used in TMS do not allow for extremely fine electromagnetic wave focusing. As a result, at least one cubic millimeter of brain tissue is resolved. Second, it is impossible to excite deeper structures without stimulating shallow structures concurrently. Nonetheless, some studies have employed TMS to boost human cognition by targeting various main information processing systems of brain including perception, learning, and memory (Manenti et al., 2012; Balan et al., 2014).

FUS is a novel experimental transcranial neurostimulation technique that uses low-intensity focused ultrasonic pulsations to induce reversible neuronal excitement or inhibition. The spatial resolution is excellent (target can be as small as 1.5 mm), and the beams have no effect on the tissues they pass through while convergent on the target point (Yoo et al., 2013). However, the procedure’s safety is still being explored, and human experimentation has only recently begun (Bystritsky et al., 2011).

Electroconvulsive treatment (ECT) is the medical delivery of a short pulse current of around 800 mA to the brain *via* electrodes attached to the temporal lobe. ECT might be regarded a sort of cognitive augmentation which is used to restore normal cognitive functions that has been compromised in mental disorders (Singh and Kar, 2017). This could occur, for example, during the acute phases of serious depression, when cognitive functions are in decline. But, a well known consequence of ECT is a brief deterioration of cognitive functions. Although the impairment is temporary and there is evidence that cognitive performance may improve in comparison to baseline levels following ECT (Hammar and Ardal, 2009; Semkovska and Mcloughlin, 2010).

#### Invasive stimulation technologies

Deep brain stimulation (DBS) is an invasive brain stimulation technique that is commonly used to treat motor diseases (e.g., Parkinson’s disease) and memory. It entails implanting neurostimulators in certain areas of the brain that emit electrical pulses to disrupt neuronal activity at the target places. Similarly, implanted electrodes are commonly used in medicine to electrically stimulate specific brain regions in order to treat uncontrollable epilepsy (Cinel et al., 2019). DBS and implanted electrodes are only employed in the medical industry to improve patient’s quality of life due to their intrusive nature, ethical concerns, and cost. As a result, research on cognitive enhancement in humans using invasive technology has been extremely limited to date, and has been limited to patients who have had devices implanted for other clinical reasons (e.g., Parkinson’s disease, epilepsy, etc.). For instance, DBS has been utilized to promote learning (Clark and Parasuraman, 2014; Suthana and Fried, 2014).

Implanted electrodes have been utilized in visual prosthesis to compensate for sensory loss in the eyes by connecting a camera to the brain *via* an electrode array implanted directly on the visual cortex. Intracortical microelectrode arrays have been utilized to transmit information from one rat’s brain to another and to boost memory recently (Cinel et al., 2019).

## Applications of Brain Augmentation and Neuroscience Technologies

**Figure 3:**
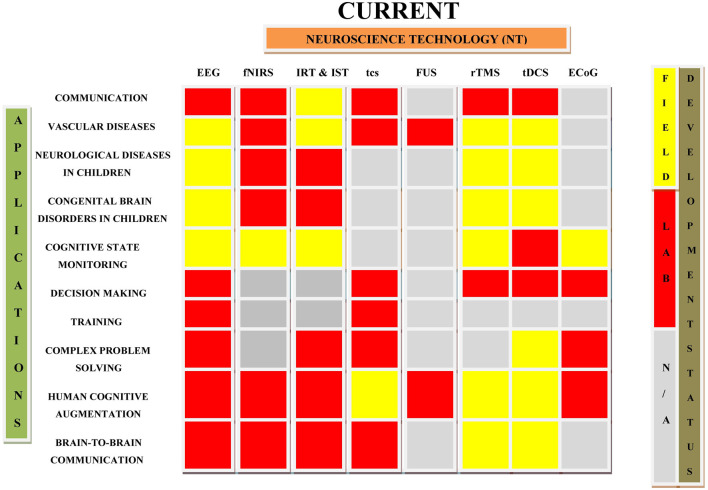
Diagrammatic representation of current applications of neuroscience technologies in various fields. This draw highlights the roadmap of the development of neuroscience technologies and their current applications in different conditions. IRT, Invasive Recording Technology; IST, Invasive Stimulation Technology (Cinel et al., 2019).

Neuroplasticity, hi-fidelity and tailored neural sensors, advanced signal processing, and machine learning techniques are all critical components of brain augmentation (Saha et al., 2021). Plenty of studies in the past showed the use of non-invasive brain augmentation for improving neurological conditions like epilepsy, stroke, Parkinson’s disease, Huntington’s disease, dementia, Alzheimer’s disease, autism spectrum disorders (ASD), traumatic brain injury, and consciousness disorders (Lebedev et al., 2018). Executive functions in ASD were improved by TMS applied to the dorso-lateral prefrontal cortex while prefrontal tDCS was found to reduce pain in multiple-sclerosis patients (Sokhadze et al., 2014; Ayache et al., 2016). Various clinical studies suggest that therapeutic non-invasive stimulation can be provided remotely under physician’s supervision, eliminating the need for patients to visit the hospital (Kennedy, 2014; Charvet et al., 2015). This section of article deals the applications of primary non-invasive neuroscience based cognitive enhancement. As invasive technologies are associated with several vulnerabilities, such as surgery-related brain tissue damage, infection, invasiveness, neuroethics, and expense, they have been seldom investigated for the treatment of disease conditions. The applications of various non-invasive and invasive neuroscience techniques in the treatment of neurological diseases, brain stimulation, communication, and in other related conditions are summarized in Table 2 and Figure 3. Table 2 lists several uses of neuroscience technologies, including the treatment of congenital brain diseases in children, brain-to-brain communications, cognitive state monitoring, complex problem-solving, and prosthetic application in the restoration of lost function.

**Table 2:** Current applications of neuroscience technologies in various fields.

**Neuroscience Technology**	**Applications**	**Reference**
**TMS**	• Stroke • Epilepsy • ADHD • Tourette's syndrome • Autism Spectrum Disorder • Depression • Congenital brain disorders in children • Cognitive state monitoring • Brain to Brain communication	• Grefkes and Fink (2020) • Fregni et al. (2005) • Weaver et al. (2012) • Kwon et al. (2011) • Baruth et al. (2010) • Walter et al. (2001) • Cinel et al. (2019)
**tDCS**	• Stroke • Vascular dementia • Epilepsy • ADHD • Autism Spectrum Disorder • Congenital brain disorders in children • Brain to Brain communication • Complex problem-solving	• Grefkes and Fink (2020) • Guo et al. (2020) • Auvichayapat et al. (2013) • Allenby et al. (2018) • Schneider and Hopp (2011) • Cinel et al. (2019)
**tES**	• Cognitive enhancement • Memory enhancement • Personnel training	• Coffman et al. (2014) • Brunoni and Vanderhasselt (2014) • Bolognini et al. (2010) • Cinel et al. (2019)
**EEG**	• Attention monitoring and enhancement • Situational awareness • Cognitive state monitoring • Congenital brain disorders in children	• Durantin et al. (2016) • Wilson and Russell (2007) • Catherwood et al. (2014) • Cinel et al. (2019)
**Prosthetics**		
• **Cochlear implants** • **Hear colors** • **Retinal Prosthesis** • **Seismic Sense** • **Magnetic Implants** • **Cyborg Nest's “North Sense”** • **Neuralink**	• Hearing restoration • Color vision restoration • Retina restoration • Experience earthquakes worldwide • Detect magnetic forces • Detect direction (poles) • Heips to operate various devices (smartphones, computers) wirelessly	• House et al. (1983) • Jeffries (2014) • Luo and da Cruz (2016) • CNN. (2018) • Robertson (2017) • Thaddeus-Johns (2017) • Musk and Neuralink (2019)
**Brain Computer Interface**	• Rehabilitation	• Anderson (2022)

### Non-invasive brain augmentation for improving brain disorders

#### Vascular diseases

Stroke is related with hemispheric dysbalance, i.e., a decrease in activity in the lesioned brain area and an increase in the activity of contralesional homologous region, limiting functional re-gain (Grefkes and Fink, 2011). As there is a close link between motor function and neural activity, manipulating brain activity could improve stroke-induced impairments. Non-invasive brain stimulation approaches can affect neuronal plasticity. TMS- or tDCS-induced changes in localized activity propagate to related brain areas, impacting activities throughout the stimulated node’s network. These approaches seem beneficial for repairing post-stroke problematic network topologies (Grefkes and Fink, 2020). In 2008, Kirton et al. (2008) conducted the first randomized sham-controlled rTMS experiment in children (median age 13.25) affected by arterial ischemic stroke showing unilateral hand motor weakness. Children received 8 days of 1 Hz rTMS of the contralesional motor cortex, which reduced inter hemispheric inhibition and promoted contralateral cortical excitability (Pal et al., 2005; Pascual-Leone et al., 2005). rTMS boosted grip strength and was also well tolerated with no notable adverse effects (Pascual-Leone et al., 2005). In another randomized, sham-controlled, double-blind, parallel clinical trial, five rTMS treatments were applied for 5 days on 17 children with infantile cerebral palsy and spastic quadriplegia. It was found that rTMS treatment caused substantial reduction in spasticity while using 5 Hz rTMS at the primary motor cortex (Valle et al., 2007). Another study also reported a significant reduction in spasticity after application of 5 Hz rTMS on the primary motor cortex that induced an overall increase in excitability of the corticospinal output system, including spinal motor neurons. Both of the trials highlighted the potential of rTMS in rehabilitating motor symptoms after childhood vascular damage (Quartarone et al., 2005).

tDCS treatment along with suitable antidepressants is found to be more efficient in treating vascular depression, resistant to antidepressants alone. Because of its non-invasive nature, lack of significant side effects, and ability to be administered to outpatients at a reasonable cost, tDCS is a valuable tool in therapeutic practice (Zanardi et al., 2020). Further studies have demonstrated a possible therapeutic role of tDCS in the treatment of cognitive impairment in Vascular dementia (Guo et al., 2020). Current evidences of neurotechnologies in the treatment of vascular disease is promising. However, due to less number of researches, tDCS has yet to be studied for the treatment of vascular diseases in children or adolescents.

#### Epilepsy

The pathophysiological feature in epilepsy is increased cortical excitability, leading to paroxysmal depolarization changes and increased frequency and synchrony of neural networks. Antiepileptic drugs by reducing neuronal excitability help to eliminate the symptoms of epilepsy (Stafstrom, 2006). Nowadays, scientists worldwide are working on use of brain augmentation approaches to treat epilepsies. In 2005, Fregni et al. (2005) examined the effect of a single 0.5 Hz rTMS session on three young patients with focal epilepsy. The TMS coil was positioned over the epileptogenic region, or in the absence of a clearly defined epileptogenic zone, over the reference point for a period of 15–30 days. The rTMS treatment dramatically reduced frequency of epileptiform discharges (ED) in the patients (Fregni et al., 2005).

In a clinical study conducted on 36 children (age 6–15 years) with focal epilepsy, it was found that active tDCS treatment significantly reduced epileptic discharge frequency at 0.24 and 48 h later. The frequency of seizures decreased slightly 4 weeks following tDCS treatment and the treatment was well tolerated (Auvichayapat et al., 2013). Researchers have suggested that tDCS can modulate the activity of epileptogenic networks. Indeed, one previous study reported that five consecutive sessions of cathodal tDCS over the primary motor cortex reduced seizure frequency and interictal epileptic discharges (IED) in patients with Lennox–Gastaut syndrome (LGS; Yang et al., 2019). Application of tDCS for 14 consecutive days significantly decreased seizure frequencies in patients with refractory focal epilepsy, with 2 × 20-min daily stimulation protocol being more effective than 20-min daily stimulation protocol (Yang et al., 2019). Use of transcranial magnetic stimulation to excite or inhibit neurons, with repetitive pulses at low-frequency producing inhibitory effects can be used to reduce cortical excitability in epilepsy (Chen et al., 2016; Walton et al., 2021). Above findings suggest that rTMS and tDCS are effective in treating epilepsy in children. However, the results are varied, presumably due to different stimulation parameters, limited sample sizes, and different etiologies of participants (Vicario and Nitsche, 2013). Since neurotechnologies are advancing at a consistent rate, they will be utilized as an intervention technique for the treatment of epilepsy, as current results are encouraging.

#### Attention-deficit hyperactivity disorder (ADHD)

ADHD is a common and debilitating illness characterized by lack of attention, hyperactivity, and executive dysfunction. Functional neuroimaging investigations have revealed hypoactivation in the cingulate, frontal, and parietal cortices in the patients of ADHD. Thus, excitation of these brain areas with non-invasive brain stimulation can be useful for ADHD (Bush, 2011). In a randomized sham controlled crossover study conducted on nine ADHD patients, 10 Hz rTMS for 2 weeks was applied on the right Dorsolateral Prefrontal Cortex (DLPFC). Both active and sham rTMS improved clinical global impression (CGI) and ADHD-IV scores with no significant side effects. As both groups in this study (those randomized to active TMS and those randomized to sham TMS) received active TMS at some point, author assessed there was an overall improvement in CGI-I in both groups across the study (CGI-I across). From baseline to the CGI-I across the study endpoint, there was a highly significant change in the primary outcome, the CGI-I across. Overall, the study participants improved by a mean of 1.1 (SD, 1.1) points (*P* < 0.005). According to author, this study was the first to explore TMS as a treatment procedure for ADHD youths. TMS was safe with no major adverse effects on research population. There was improvement in the symptoms of the patient from the start to the finish of the trial but there was no difference between treatment conditions. The improvement with sham TMS in the initial phase of the trial made it impossible to determine TMS’s efficacy, albeit 95% confidence intervals suggest clinically significant effects. Since this was an exploratory study, the results were promising (Weaver et al., 2012).

tDCS of DLPFC has been shown to modulate cognitive circuits and could enhance DLPFC activity, leading to improved impulse control in ADHD (Allenby et al., 2018). Researchers have shown that tDCS can reduce the symptoms of ADHD in adolescents and enhance their cognitive performance (Soff et al., 2017). However effect of tDCS on ADHD in children is still a subject of research (Allenby et al., 2018).

rTMS is also known to promote the secretion of dopamine by stimulating the prefrontal lobe of the brain, thereby improving the symptoms of ADHD (Bloch et al., 2010). rTMS is an efficacious intervention for treating ADHD, and combined rTMS and atomoxetine is superior to atomoxetine alone in improving attention deficit symptoms and total ADHD symptoms severity (Nagy et al., 2022). However the rTMS treatment of ADHD is still in its infancy and we need to focus on best treatment regimen, duration of acute treatment, neurostimulation target, and symptoms modulated by rTMS in ADHD (Kumar et al., 2019). As current study results are encouraging, these neurotechnologies needs to be investigated in future for the treatment of ADHD.

#### Tourette’s syndrome

Tourette’s syndrome (TS) is a prevalent pediatric neurobehavioral condition characterized by fast tics, stereotyped movements and vocalizations that affect practically all body segments. The striatum, sub-cortical areas and Supplemental Motor Area (SMA) are impaired in childhood TS (Swain et al., 2007; Vicario et al., 2010). Patients with TS have a hyperexcitable premotor cortex (George et al., 2001). Because SMA is mainly related to regions involved in TS, excitability-reducing rTMS to the SMA may be an effective treatment for TS (Picard and Strick, 2001). In a 12 week cohort pilot study, 1 Hz rTMS was applied across the SMA of patients each day and mood, anxiety level, tics, and side effect were monitored. It was observed that the treatment statistically decreased Yale Global TS Severity Scale (Kwon et al., 2011). In another study, application of 1 Hz rTMS on the SMA in 25 TS children for 20 days resulted in considerable improvement in symptoms of TS. Intriguingly, 68% of participants reported improvements lasting up to 6 months (Le et al., 2013). However, no tDCS researches for TS have been done so far. Preliminary evidence suggests that tDCS may be useful in the treatment of Tourette syndrome. Larger scale studies are required to ascertain the improvement of symptoms over time, as well as the long-term consequences of the repetitions of sessions (Eapen et al., 2017; Kleimaker et al., 2020). Based on current findings, the authors are optimistic about the success of neurotechnologies in the treatment of TS in future.

#### Autism spectrum disorder (ASD)

ASD affects around 1 in 150 children worldwide and causes significant social and communication deficits. Non-invasive brain stimulation has been found to improve the symptoms and cognition in the patient of ASD (Fombonne, 2005). Increased gamma-band responses to several cognitive processes in children with ASD have been described (McFadden et al., 2012). In a controlled investigation conducted on 25 patients of ASD (ages 9–26) and 20 age-matched controls, the electrophysiological effects of 12 low frequency rTMS sessions (weekly application) applied bilaterally to the DLPFC (first six treatments over the left DLPFC and the rest over the right) was studied. After the exposure period, discriminatory gamma activity and behavioral characteristics improved significantly in the treatment group (Baruth et al., 2010). In an another study conducted on 20 ASD patients (ages 10–19) using oddball paradigm (García-Larrea et al., 1992) to examine attentional shifting, rTMS caused significant reduction in mistake percentage, repetitive-ritualistic behavior, and early cortical reactions to irrelevant stimuli (Sokhadze et al., 2010).

Similarly in a study, tDCS was applied in autistic youngsters (10 from age group of 16–21) who had limited verbal communication. It was found that post-anodal tDCS of the Broca region increased mean vocabulary scores compared to pre-anodal tDCS (Schneider and Hopp, 2011). Therefore research indicates that non-invasive brain stimulation may be beneficial to autistic youngsters. However, detail studies are still lacking on the subject (Vicario and Nitsche, 2013).

#### Depression

Childhood depression is associated with considerable functional impairment in various domains and areas of brain (Cosgrove et al., 2013). In addition to the left dorsolateral prefrontal cortex, the subgenual cingulate gyrus is also affected in depression (Mayberg, 2007). It has been demonstrated that in depression, hypoactivation of left hemisphere and hyperactivation of right hemispherical areas takes place resulting in a hemispheric imbalance. With the help of brain stimulation technique we can increase the activity of left dorsolateral prefrontal cortex (Fitzgerald et al., 2006). In 2001, Walter et al. studied the effect of rTMS on depression in three patients below the age of 18. The patients underwent daily treatment with 10 Hz rTMS over the left DLPFC for 2 weeks. Clinically, two subjects benefited, while one experienced tension headaches throughout the period of treatment sessions (Walter et al., 2001). In an another study, application of 10 Hz rTMS on the left DLPFC in depressed and ADHD children for 6 weeks improved symptoms of depression but no improvement was observed in the symptoms of ADHD (Loo et al., 2006; Bloch et al., 2008). In a similar study conducted on eight treatment-resistant adolescents, application of 10 Hz rTMS on the left dorsolateral prefrontal cortex daily for 30 days showed significant improvement in Children’s Depression Rating Scale–Revised mean score. Neurocognitive testing also revealed no deterioration of functions pre- or post-treatment (Wall et al., 2011). Although rTMS is a recognized treatment for Major Depressive Disorder (MDD), the therapeutic area is constantly advancing to improve response and remission rates. Novel stimulation parameters and fine-tuning personalized therapy methods are being studied. Despite this, significant unsolved concerns remain, such as the stability of medium to long-term antidepressant benefits of this technology, appropriate sequencing between rTMS sessions, conjunction with other antidepressant treatments, or the hypothesized role of rTMS as a cognitive enhancer (Baeken et al., 2019). In light of the above mentioned study findings, the authors are optimistic about neurotechnologies’ efficacy in treating depression.

#### Schizophrenia

Childhood-onset schizophrenia is a severe type of the condition that shares many similarities with adult-onset schizophrenia, hallucinations being the most distressing clinical symptom (Nicolson and Rapoport, 1999; David et al., 2011). The right medial temporal, lateral temporal, inferior frontal cortex, cingulate cortex, left DLPFC, and left superior temporal gyrus all shows physiological abnormalities in schizophrenia (Vyas and Gogtay, 2012). Auditory hallucinations in schizophrenia are connected with increased left temporoparietal cortical excitability (Silbersweig et al., 1995). However, increased activity in the left prefrontal region may help alleviate negative symptoms by increasing dopamine release (Heimer et al., 1997). Excitability-reducing stimulation may diminish left temporoparietal cortex activity, whereas excitatory non-invasive brain stimulation may enhance left prefrontal cortex activity (Freitas et al., 2009; Demirtas-Tatlidede et al., 2013). Recently the effect of tDCS was studied on 12 children with schizophrenia in a randomized controlled trial. The patients were randomly assigned to either bilateral anodal DLPFC stimulation (*n* = 8) or bilateral cathodal superior temporal gyrus (STG) stimulation (*n* = 5) and the treatment was given for 20 min every day for 2 weeks. As such no discomfort was experienced by the subjects and only four subjects suffered temporary redness under the electrodes that vanishes after hour of treatment. Although no significant clinical improvement has been reported; this study was the first to show that tDCS with the applied settings is well tolerated in adolescents (Mattai et al., 2011). However, the benefits of non-invasive brain stimulation in childhood onset schizophrenia are largely unknown. The only tDCS study available supports this stimulation protocol’s tolerability (Vicario and Nitsche, 2013). The authors hope that substantial amounts of research need to be conducted before these neurotechnologies emerge as a treatment for schizophrenia.

#### Brain augmentation for rehabilitation

Brain prostheses are intended to be artificial systems directly connected to the brain in order to replace a damaged area or connect disconnected regions and restore lost functionality. For instance, the device may be used to reconnect somatosensory and motor cortical areas to restore forelimb movement following a brain injury. In 2013, Kansas University Medical Center presented for the first time a brain prosthesis with an architecture implementing a closed-loop reactive policy (Guggenmos et al., 2013). An additional promising example is the hippocampal memory prosthesis, in which the neural activity of specific hippocampus regions that have been suitably processed can be used to manipulate and thus restore (*via ad hoc* electrical stimulation) cognitive mnemonic processes. Brain prostheses are still in the preclinical stage of development (Panuccio et al., 2018).

The work of Courtine and colleagues, which successfully demonstrated that spatiotemporal spinal cord modulations can restore locomotion in spinal cord-injured rodents, lies at the boundary between BMIs and brain prostheses. Notably, all of the above mentioned neuroprosthetic devices take advantage of recent software and hardware advancements in terms of the amount of data processing (Wenger et al., 2014, 2016). For instance, optogenetics and sonogenetics enable precise spatiotemporal control of cells and the manipulation of specific brain circuits *via* light and sound, respectively. Therefore, these techniques can be utilized in future neuroprosthetic devices, which has significant implications for the treatment of neurological disorders (Panuccio et al., 2018).

### Brain augmentation in communication

Recording based non-invasive BCI systems often detect certain patterns of brain activity and transform them into device commands or communication acts (Cinel et al., 2019). Among EEG-based BCIs, Event-Related Potentials (ERPs), or series of oscillations in the electrical signals that are recorded from the scalp in response to abrupt sensory, cognitive, or motor events is a major focus of the study (Luck, 2005). Slow Cortical Potentials (SCPs), Mu wave-Related Desynchronization, mental imagery, and Steady-State Visually Evoked Potentials (SSVEPs) are few brain augmentation techniques that are more frequently used in communication. SCPs, Event-related desynchronization (ERDs), and BCIs based on mental imagery are fundamentally biofeedback-based and are not dependent on external stimuli in the way that ERP- and SSVEP-based BCIs are (Elbert et al., 1980; Birbaumer, 2006; Pfurtscheller and Neuper, 2006; Amiri et al., 2013). Invasive recording methods have been applied in several sorts of augmentation technologies, resulting in greater performance/ITR than non-invasive counterparts (Tehovnik et al., 2013; Baranauskas, 2014). Because electrode implantation poses medical and ethical issues, most invasive BCI research has been done on monkeys or rats, with human studies being rare (Chapin et al., 1999; Taylor et al., 2002; Borton et al., 2013). Paralyzed people can also utilize BCIs with implanted electrodes to speak instead of write. BCI predicts intended speech directly from neural activity. This data is used to control a voice synthesizer. With advancement in science, researchers have been investigating the idea of direct brain-to-brain communication, i.e., physically joining brains to allow direct information flow. Pais-Vieira et al. (2013, 2015) successfully tested this in rats, where an encoder rat was trained to accomplish a task that was then “communicated” to a decoder rat. The encoder rat’s motor cortex synaptic activity was recorded invasively and then sent to the decoder rat through invasive intracortical micro stimulation so that the decoder rat might learn the same task (Pais-Vieira et al., 2013).

### Brain augmentation in perceptual optimization

This section focuses on applications of brain augmentation for individual and group decision making, as well as cognitive enhancement based on brain stimulation.

#### Decision-making

How people make decisions individually and collectively is a subject of research since ancient times. Several processes and mechanisms influence decision-making, including early perceptual processes, attention, and working memory processing (Cinel et al., 2019). Recent brain research has focused on how people make decisions, their methods, and their willingness to take risks (Rushworth and Behrens, 2008; Tobler et al., 2009). With so much neuroscientific knowledge about information and decision processing, it seems reasonable to try to improve decision-making. However, the most viable non-invasive sources of information on brain activity like tES and TMS are exceedingly noisy, making it extremely difficult to provide information about (or assist with) individual decisions with any degree of confidence (Cinel et al., 2019).

#### Brain augmentation for cognitive enhancement

Techniques like tES and TMS can increase perception, learning, memory, attention, and decision-making (Coffman et al., 2014). Several researchers have showed proven ways to improve target detection (e.g., visual search) and tracking with the help of non-invasive brain augmentation. Anodal stimulation enhanced performance slightly. tES also performed well in a more realistic and complex threat detection scenario where participants were shown a short video clip from a virtual reality setting and asked to determine whether a threat was there. tES dramatically enhanced performance in both studies (Clark et al., 2012).

In one tDCS trial, participants were shown a display of simple, colored shapes and asked to decide whether or not a target was present (Nelson et al., 2015). tDCS has also been used successfully to treat reading difficulties such as dyslexia in both adults and children (Heth and Lavidor, 2015). However, the benefit of tDCS on reading appears to be limited to certain activities, such as sight word efficiency (Younger et al., 2016). Hence, brain stimulation may be utilized to optimize cortical oscillations, resulting in indirect improvements in a variety of tasks (e.g., stimulus binding; Horschig et al., 2014).

#### Brain augmentation in memory enhancement

Numerous studies have shown that non-invasive brain stimulation using TMS and tES improves memory and learning (Brunoni and Vanderhasselt, 2014). The N-Back and Sternberg tasks, for example, have shown that tDCS can help both healthy and memory-impaired people to acquire sequential motor sequences and complicated motor patterns (Cinel et al., 2019). A number of studies suggest that the advantages of tES/TMS stimulation on short- and long-term memory can last for 4–6 weeks following stimulation (Ohn et al., 2008). Invasive neurotechnologies have also showed promise in memory enhancement. Recent achievements include neuroprosthetics that improve memory encoding and retention. They use nonlinear systems technique to compute multiple-input/multiple-output (MIMO) connections, where inputs are spike trains from CA3 neurons generating output spike trains in CA1 (Berger et al., 2005, 2010).

#### Attention monitoring and enhancement

Numerous studies and technologies are focused at real-time monitoring of cognitive function and capacity, such as working memory capacity or attention. Even when such systems were not explicitly designed to improve performance, monitoring the mental state of users enables performance to be improved by altering the interface with which they interact, referred to as adaptive interfaces. In 2007, Wilson and Russell (2007) proposed a neuroadaptive system in which users were given a task to detect a target in their environment and whose mental effort was varied in response to input provided by EEG and other physiological indicators (Durantin et al., 2016). In general, as alertness decreases, low-frequency EEG oscillations and ERP amplitudes increase. Changes in EEG patterns (increased theta and decreased beta activity) associated with the awake-sleep transition might potentially suggest attention deficits. Additionally, it is known that the amplitude of the P300 ERP is connected to mental strain and the level of attention for a particular task. This has also been demonstrated in complex aviation and driving simulation scenarios, where the P300 can provide a workload assessment (Cinel et al., 2019). The use of BCIs as a form of biofeedback to improve visual attention has been investigated (Strehl et al., 2017). Although it has only been tested on ADHD individuals, neurofeedback has also been demonstrated to help tinnitus patients focus on the auditory perceptual modality (thereby giving them the ability to suppress or reduce the effects of tinnitus; Busse et al., 2008).

#### Situation awareness

Situational awareness is the ability to perceive, know, and grasp the current state of complicated, dynamic situations and being aware of what is going to be important to the task or goal at hand. Situation awareness can be studied in military command and control, combat aircraft, air traffic control, emergency services, and other domains where information flow is high and errors might be catastrophic (Endsley, 1995). Recent research shows that neurophysiological approaches can be used to examine cognitive processes linked with situation awareness in military simulations (Berka et al., 2005). Perception of environmental factors or stimuli can be measured using 128-channel EEG while identifying targets and threats in urban situations. In both cases, the target was abruptly changed, causing confusion. In the 100–150 ms following the loss of situation awareness, there is co-activity in ocular, prefrontal, anterior cingulate, and parietal regions linked to cognition (Catherwood et al., 2014). Portable EEG equipment can also monitor situation awareness in air traffic controllers in real-time (Yeo et al., 2017).

#### Hyperscanning

Hyperscanning is a technology that records the brain activity of two or more people working together on a common goal (Babiloni and Astolfi, 2014). Currently, hyperscanning is employed to find the correlation of brain activity between people. Studies utilizing hyperscanning have found some of the neural correlates of interaction in two brains, and recorded how they alter as the players come to know each other and their engagement during the game evolves. The technology is not yet employed for communication, cognitive enhancement, or social engagement. However, in the near future, this technology may improve such tasks (Cinel et al., 2019).

#### Personnel training

Brain-based training has recently gained popularity in the security and defence industries. They may improve training by allowing it to be tailored to the needs of individual users rather than employing a one-size-fits-all approach (Stanney et al., 2011). Technology can improve training by monitoring brain activity (Miranda et al., 2015). tES can be utilized to increase visual search and exploration task learning (Bolognini et al., 2010). In a study it was found that tES enhances target acquisition accuracy and speeds up acquiring threat detection skills (McKinley et al., 2013).

#### Complex problem-solving

Neuroscience technology can also help with problem-solving. It was found that tDCS could boost performance in the Remote Associates Test, a verbal problem-solving activity requiring participants to accurately estimate three cue words linked by a fourth word (Cerruti and Schlaug, 2009). Another study showed that tDCS improved 40% of participants’ ability to complete a challenging puzzle (Chi and Snyder, 2012). tDCS based problem-solving augmentation also increased speed and accuracy of a given task (Dockery et al., 2009).

## Risk Factors with Brain Augmentation and Neurotechnologies

Augmentation technologies are designed to enhance human skills and continuous development of neuroenhancement technologies may lead to increasingly more helpful forms of brain augmentation. However its eventual utility is debated, most evidence suggests that future research will lead to at least a few beneficial applications, if not many. Many people believe that some forms of neuroenhancement are too uncommon, immoral or unethical and should be outlawed or carefully regulated (Walsh, 2013; Clark, 2014; Shook et al., 2014). Brain augmentation technologies have various limitations or drawbacks.

1. Cognitive augmentations may benefit primarily the wealthy due to high pricing, hence deepening the social and cultural divide generated by income disparities (Hyman, 2011).
2. Cognitive augmentation may result in a “arms race” of enhancement, in which everyone is compelled to utilize enhancement in order to remain competitive (Hyman, 2011).
3. Improving one cognitive domain may result in a decline in another. This argument assumes that emphasizing one sort of cognition must necessarily diminish others (Luber and Lisanby, 2014). The enhancement issue in neuroscience and biomedical ethics tends to focus on memory, learning, and attention augmentation. Typically, the point of argument is whether these augmentative upgrades should be permitted for individuals who do not have a specific “medical” deficit along any of the dimensions of interest? (Earp et al., 2014).
4. Uncertainty about safety is also a concern. No brain stimulation technology is without side effects. The risks and benefits of surgical techniques like DBS are carefully weighed against the potential risks and benefits to the patient. There are actual hazards of seizures from TMS and tCS, and scalp burns from tCS, even though the safety limits for brain stimulation are well defined (Davis and van Koningsbruggen, 2013).
5. Expanding access to stimulation of the brain. Low cost and ease of fabrication of tDCS has led to a surge in “DIY-tDCS” for self-stimulation. Ethical and policy consequences of augmenting technology are also an issue (Davis and van Koningsbruggen, 2013). In addition, persons who are dissatisfied with current drug treatment seek supplementary treatment with brain stimulation, without clear instructions concerning necessary controls or combinations with existing treatments (Davis et al., 2013; Cabrera et al., 2014).

## Neuroscience Technologies for Brain Augmentation: Challenges and Hindrances

Current research suggests that among all neuroscience techniques tDCS can mainly improve cognitive and behavioral performance. These enhancement claims are likely to be the driving force behind the recent rise in public interest in this technology. The three variables that can be adjusted to improve tDCS efficacy are current density, electrode position, and stimulation time. While these three variables clearly influence tDCS outcomes, there are a number of equally critical problems related to efficacy and mechanism that are just not being addressed (Horvath et al., 2014). While Human Performance Enhancement Technology (HPET) has demonstrated some benefits in some areas, its deployment may result in health and safety concerns, and even death, among augmented individuals (Shao et al., 2021).

### Inter-subject variability

Before it may be used in healthy or clinical populations, tDCS must show equivalent results across a wide variety of persons. In fact, extensive between- and within-group heterogeneity suggests an uneven influence amongst individuals (Horvath et al., 2014). Even two groups receiving same stimulation procedure (0.0286 mA/cm^2^ current density; anode M1/cathode contralateral orbit montage; 5 min duration) showed considerable intergroup variation after 5 min of tDCS treatment with one group showed 93.2% increase in MEP amplitude, whereas the other showed only a 9.2% increase (Fricke et al., 2011). Modern MRI guided neuronavigation systems can ensure correct coil location over time, although many tDCS studies do not use them. As a result, slight changes in coil placement and orientation over time may affect response variation (Herwig et al., 2001; Ahdab et al., 2010). Neurophysiology, anatomy, and psychology may influence tDCS response. Recent research reveals that characteristics including skull thickness, subcutaneous fat levels, cerebrospinal fluid density, and cortical surface topography can considerably influence current flow and density patterns during stimulation (Datta et al., 2012).

### Intra-subject reliability

Before this technology can be used effectively, it must be proven that people respond consistently to repeated tDCS sessions as individual response reliability of tDCS has not been examined yet (Horvath et al., 2014). Circadian, metabolic, and hormonal cycles may influence responsiveness. In fact, multiple studies have indicated that menstrual cycle stage and cortisol levels influence plastic responsiveness to TMS treatments (Smith et al., 1999; Sale et al., 2008).

### Motor and cognitive interference

Several lines of studies indicate that active motor and/or cognitive engagement during tDCS can reduce or eliminate its effects (Antal et al., 2007). When combined with motor training, the tDCS benefit may be lost. Neurologists often instruct participants to relax during long-duration off-line stimulatory techniques. Relaxation can be achieved through reading, messaging, perusing the internet, or completing chores. It is critical to determine the effects of tDCS in normal human behavior before wasting time and money on ineffective protocols. Until this is rectified, practitioners should limit motor and cognitive activity during and soon after tDCS, as well as any subsequent procedure (TMS, MRI, etc.; Horvath et al., 2014).

### Electric current influences

Variables that may greatly influence current density and flow can also affect the effectiveness of augmentation techniques (Paulus, 2011). Hair thickness as well as condition can influence current density and flow. Past investigations suggest dry hairs (<7% 25% H_2_O content) have a resistivity of approximately 3 × 10^12^ Ω/cm while wet hairs (25% H_2_O content) have a resistivity of approximately 6 × 10^6^ Ω/cm (Feughelman, 1997). Less resistance equals more conductivity. To combat this, practitioners frequently saturate dense hair with saline. Unfortunately, saline or drip- ping on the scalp might cause unwanted and unpredictable current flow. The tDCS current can be bridged even when there is no direct electrode-to-scalp contact (such as in participants with thick hair). The exact location of the electric current entry and departure locations on the scalp will thereafter be unknown and unpredictable. Also, when an electric current travels *via* saltwater to the scalp, the current density is unknown and unexpected (Horvath et al., 2014). Sweat can influence electric current dynamics and increases skin conductivity, hence affect current flow. The epidermis may generate enough conductivity as salts and oils collected in scalp pores can prevent current from entering the cortex (Dawson et al., 2017). Finally, the way electrodes are attached to the scalp may affect current dynamics and flow. For example, many modern tDCS sponge electrodes have plastic rings around the corners. Unless carefully designed, most polymers are non-conductive. Although it is unknown whether or not the plastic that was used to make these electrodes was produced to conduct electricity (Horvath et al., 2014).

### Additional issues

Recent advances in optogenetic stimulation technologies pose ethical problems, not only about the appropriateness of brain interventions and their repercussions, but also about the necessary genetic alterations of the organism. To make mild stimulations conceivable, modified viruses must be used to remodel cells. This requires more than just weighing risks and rewards. The question is whether the benefits of optical stimulation over electrical stimulation (which causes irreversible damage to neural tissue) can be offset by the risks of genetic alteration (Müller and Rotter, 2017).

## Ethical Issues with Neuroscience Technologies

Natural forces balance human physiological, pathological, and psychological changes, leaving the organism in an overall healthy state (Brown and Tvaryanas, 2008; Lin and Allhoff, 2008). It is natural to fear changes and the unknown. Thus, the ethical discussion often appears to center on what is imaginable, rather than what is scientifically foreseeable and what is now actuality. This can lead to unforeseen outcomes (Cinel et al., 2019). Along with the more classic ethical difficulties relating to human engagement in research, advances in neuroscience and neuroscience technologies have produced new and unique ethical issues (neuroethics; Chan and Harris, 2012; McCullagh et al., 2014). Ethical concerns regarding brain-augmentation methods include the relationship between diminishment and enhancement following the application of brain-augmentation technologies. The obligation to use cognitive enhancers in high-risk professions, determining the population who are in need of brain enhancement, informed public policy, cognitive biases, and the hype generated by the development of brain-augmentation technologies are other concerns (Lebedev et al., 2018). It is difficult to anticipate the future of neuroscience, neuroergonomics, BCIs, and human enhancement technologies, and also impossible to foresee how society will view them. Tracking ethical implications is vital, especially in domains like mind reading, privacy, agency, accountability, and liability. Since BCIs, neuroergonomics, neural engineering etc. are gaining prominence in the field of neuroscience, such applications warrant ethical consideration (Cinel et al., 2019).

Ethical concerns regarding mind reading and privacy include the ability of neuroimaging techniques like EEG or fMRI to detect, map, and interpret an individual’s brain activity in specific situations. Due to their ability to “read” or otherwise “assess” someone’s thoughts, emotional states or attitudes, such approaches may raise problems about free will and privacy (Chan and Harris, 2012). Mind reading is a concern in conditions where mental activity is tracked, such as in neuroergonomics, passive BCIs, or hyperscanning (Trimper et al., 2014). Various researchers have further raised the problem of accountability with BCI and other brain augmentation techniques. Who will be responsible for actions taken by the decoder when the encoder’s brain is connected to the decoder’s brain? Understanding agency, accountability, and responsibility will become increasingly difficult as the amount and complexity of possible messages conveyed to decoder increases (McCullagh et al., 2014; Lebedev et al., 2018).

Various people worldwide question on safety and invasiveness of brain augmentation techniques and assume that they can alter brain activity. One must be sure and ask whether intrusive procedures such as DBS are safe or safer than other ways currently in use when considering neurostimulation technologies from an ethical perspective (Clark, 2014). tES and TMS (frequently utilized for cognitive enhancement) are associated with issue of invasiveness though they are non-invasive kinds of stimulation (Davis and van Koningsbruggen, 2013). Another problem is the costs vs. advantages of neuroscience technologies for society. Aside from potential hazards, it is often uncertain if these technologies are beneficial to society. Increasing reliance on neuroscience technologies may have unintended negative societal consequences (Cinel et al., 2019). While expectations are crucial in advancing science and technology, but they are not always accurate forecasters of the future (Brown and Michael, 2003). Even though a wide multidisciplinary community of scientists shares high expectations for future breakthroughs, this does not guarantee scientific success of brain augmentation techniques (Pollock and Williams, 2010). When expectations are not met, enthusiasm for planned scientific development wanes which possibly is followed by a gradual resurgence. This is referred to as a “hype-disappointment cycle” (Van Lente et al., 2013). Unrealistic expectations might have a negative effect that is why neuroscientists should avoid overhyping the potential for brain augmentation (Rusconi and Mitchener-Nissen, 2014). The authors are of the opinion that as it is difficult to predict the precise future trajectory of neuroscience, BCIs, and brain augmentation technologies, it is similarly difficult to predict neuroethics, i.e., how society will view these technologies. It is crucial to monitor ethical implications, especially in areas such as mind-reading and privacy, agency, responsibility, and liability. Given the recent trajectory of neuroscience, BCIs, brain-to-brain communication, and neural engineering, as well as their tremendous growth, such applications have potential to become a reality 1 day; therefore, they deserve ethical discussion.

## Current Status of Neuroscience Technologies for Brain Augmentation

Neurotechnologies are combination of neuroscience and engineering that enable the research, repair, and enhancement of brain function. Non-invasive brain imaging techniques such as EEG and fNIRS have become increasingly accessible and affordable over the last few decades, paving the way for novel applications of neurotechnologies (Ayaz and Dehais, 2018). Neuroergonomics and neural engineering have recently emphasized the use of non-invasive neurotechnologies to increase a variety of human capacities, including communication, emotion, perception, memory, problem-solving, and decision-making (Kosmyna and Maes, 2019). Figure 3 depicts current applications and development status of various brain augmentation techniques. Noninvasive brain stimulation techniques like TMS and tES are employed in the investigation, prognosis, and treatment of a wide variety of illnesses (Bikson et al., 2020). Direct brain manipulation on a targeted basis may also be accomplished *via* visual sensory substitution and somatosensory senses (Adaikkan and Tsai, 2020). TMS has been cleared by the FDA for the treatment of serious depression and obsessive-compulsive disorders. Other non-invasive neurotechnologies have been used to monitor speaker-listener interaction, decode participants’ mental states, and facilitate brain-to-brain communication between numerous brains (Gaudry et al., 2021).

Among invasive neurotechnology, ECoG is a well-established version of such technology (Neely et al., 2018). Although invasive technologies are linked with numerous dangers (e.g., brain tissue damage related with surgery, infection, etc.), they currently offer the greatest portability and the quickest operation (Hendriks et al., 2019). Improved non-invasive technologies become more competitive, with the aim of enhancing perceived benefits in relation to associated dangers. Various studies are focused at decoding certain mental states and speech using non-invasive neuroimaging (Anumanchipalli et al., 2019). Convolutional neural networks are available with 90% accuracy in learning as compared to classical machine learning. These findings demonstrate the prospects of deep learning in neural decoding for human enhancement (Asgher et al., 2020). Scientist worldwide are working on brain implants to improve their long-term stability and biocompatibility so that they can be used outside of clinical trials. Engineers are focusing on modern robotics to increase the precision and dependability of neuroprostheses for patients, using machine learning to make them adaptive and “intelligent”. Along with “readout” electrodes, stimulating electrodes are implanted into the brain to activate or inhibit specific nuclei, regions, or fiber bundles externally *via* electric current or, more recently, light. However light stimulation techniques, recommended for improving hearing aids or focusing inhibitory “counter steering” in epileptic seizures have serious drawbacks (Müller and Rotter, 2017). New treatments for debilitating brain illnesses are anticipated to emerge in the longer term as a result of a better understanding of the brain. For example, circuit-level understanding of the brain’s motor systems has significantly aided in the treatment of Parkinson’s disease (Cerasa et al., 2014). As a result, teams of neurophysiologists, engineers, and physicians can work together to develop deep brain stimulation, which can restore motor circuit function in many Parkinson’s patients for several years (Schuepbach et al., 2013). Current research into mood and emotion-related brain circuitry also has the potential to revolutionize psychiatry in a similar manner (Holtzheimer and Mayberg, 2011). Brain mapping is already an exciting subject of science. Brainbow and CLARITY are two new anatomic techniques that reveal unprecedented views of neural architecture (Chung et al., 2013). Further, innovative technologies—including two-photon imaging, light sheet microscopy, and miniature microendoscopes—along with calcium imaging and voltage imaging—have enabled us to gain the first dynamic views of how the brain encodes information in modular circuits (Ahrens et al., 2013). Optogenetics has enabled precise manipulation of circuit activity with light pulses (Tye and Deisseroth, 2012).

### Brain 2025: a scientific vision

Recognizing that modern neuroscience is on the verge of revolution, American President Barak Obama launched the Brain Research through Advancing Innovative Neurotechnologies (BRAIN) Initiative as a bold new research effort aimed at “giving scientists the tools they need to obtain a dynamic picture of the brain in action” (Obama, 2013). Aware of the enormity of this aim, the President called on all stakeholders to join the BRAIN Initiative, including companies, health systems, patient advocacy groups, philanthropists, state governments, research universities, and private research institutes. A better knowledge of how the brain generates complex ideas and behaviors would help progress in identifying, treating, and possibly curing neurological and mental diseases and disorders that wreck so many lives (Jorgenson et al., 2015; Mott et al., 2018). The BRAIN Initiative’s most significant outcome will be a comprehensive, mechanistic knowledge of brain functions that results from the synergistic application of the new technologies and conceptual structures produced through the BRAIN Initiative (Jorgenson et al., 2015; Greely et al., 2018). The United States commitment will be significantly bolstered by the growth of complementary global programs, such as the European Union’s Human Brain Project, Japan’s Brain/MINDS (Brain Mapping by Integrated Neurotechnologies for Disease Studies) project and Canada Brain, are few to mention (Markram et al., 2011; Okano et al., 2015). Additionally, China is planning a national brain project (Poo et al., 2016). Hence, it will be critical for the research community to maintain a consistent dialogue regarding the scientific opportunities and challenges inherent in these large-scale projects (Jorgenson et al., 2015). The Human Connectome Project has boosted gradient strength and enhanced white matter imaging to create the first detailed “wiring diagram” of the living human brain, providing fresh insights into the three-dimensional architecture of fiber tracts (Wedeen et al., 2012). fMRI advancements have provided us with more accurate maps of human brain activity, enabling for more exact localization of complex activities such as language, emotion, decision-making, and hallucinations (Schölvinck et al., 2010).

## Future Prospects of Brain Augmentation and Neuroscience Technologies

**Figure 4:**
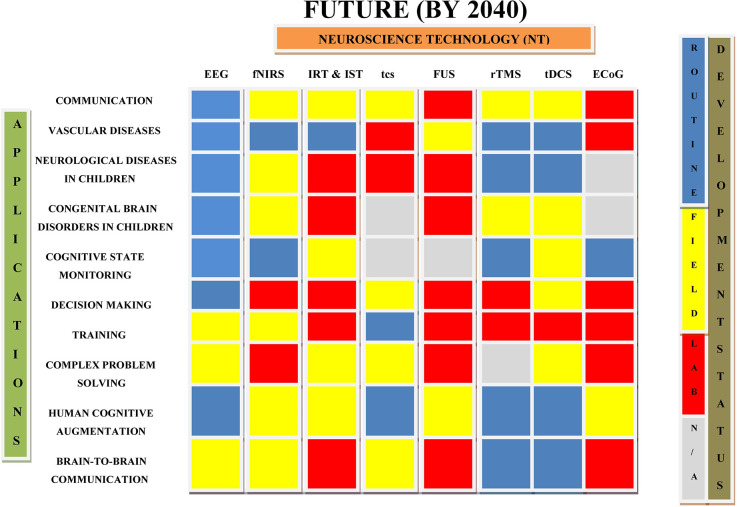
Future prospects of neuroscience technologies. The picture highlights future development and applications of neuroscience technologies for different medical and non-medical purposes (Cinel et al., 2019).

This section deals with long-term prospects for augmentation techniques that have only recently made the transition from science fiction to scientific theory and investigation. Figure 4 illustrates the predicted future of various non-invasive and invasive brain augmentation techniques.

Advancements in brain machine interface technology may be able to assist humanity in dealing with the “moment of singularity,” when artificial intelligence will exceed human intelligence (Kennedy, 2014; Lebedev et al., 2018). Within the roadmap’s prediction horizon (more than two decades), advancements in brain augmentation are likely to accelerate, particularly as ethical, medical, and technological barriers are gradually reducing, paving the way for the viability of intrusive brain activity observation technologies. In general, it is envisaged that BCIs for communication and control have advanced sufficiently to used widely, particularly in sectors where reaction times greater than those of the musculoskeletal system are necessary or covert communication is required. However, it is also obvious that many neurological technologies for enhancing human performance will continue to transfer outside the lab for field testing, with some even utilized in normal use over this time period (Dresler et al., 2013). Non-invasive brain stimulation to children may provide benefits superior than those achieved in adults. Thus, non-invasive stimulation treatment may be more reliable and constant in children. According to previous research, this same “sensitivity” may cause maladaptive brain plasticity (Knudsen, 2004). To mitigate this risk, future study should focus on investigation of the physiological effects of non-invasive brain stimulation in children. Systematic conduct of “dose-finding” sham-controlled, double-blinded experiments will provide critical information about the safety and effectiveness of stimulation techniques. These characteristics will aid in clarifying the potential therapeutic efficacy of non-invasive brain stimulation in children at multiple levels of complexity, providing a realistic basis for large-scale clinical implementation of such stimulation regimens (Vicario and Nitsche, 2013).

### Future of neuroscience technologies for recording and stimulating brain activities

Given the benefits of each neuroscience technology, it seems likely that each will continue to evolve over the next two decades. In terms of current and future uses, EEG and fNIRS may be the ideal techniques due to their portability, low cost, non-invasiveness, and extensive use in BCI and neuroergonomics studies. If dry electrode technology continues to advance at its current rate, EEG may become even more realistic (Cinel et al., 2019). As long as the risks associated with the presence of electrodes inside the body are less, invasive brain activity surveillance techniques like ECoG or implanted electrodes will become increasingly ethical and medically acceptable. However, invasive procedures may be more accurate and successful in viewing brain activity, if current advancements in recording technology continue (Pesaran et al., 2018). In terms of neurostimulation technologies, the best advantages are offered by tES, which is portable, generally inexpensive, and non-invasive. The recent development of a higher resolution variant of tES indicates that further advances are on the way Eventually, FUS may outperform both technologies in terms of resolution and portability, but it is unknown if it will ever be viable to simultaneously stimulate many sites and huge portions of the brain. It is obvious that invasive approaches, such as implanted electrodes, will provide a more direct and precise means to influence brain activity (Cinel et al., 2019).

The authors are optimistic about the future of brain augmentation based on the present pace of advancement in neuroscience technologies. In contrast, brain augmentation is a fascinating and interesting scientific marvel, and as the saying goes, “Nothing interesting is ever completely one-sided.” As society becomes more exposed to neuroscience technologies for brain augmentation, one would anticipate a gradual shift in ethical thought, resulting in an acceleration of their development and application. However, as neurotechnologies develop, the need for unambiguous ethical governance grows more pressing.

## Conclusion

Just as smart phones and the Internet changed how we lived 20 years ago, brain machine interfaces 20 years from now, may enable more intimate and direct collaborations between brains and technology, enabling enhancement of sensory, motor and cognitive skills, communications and can help in treating various neurological conditions. Neuroscience technologies can be a “last resort” treatment for many disorders due to which patients have lost their employment and social connections. Recent research and funding priorities indicate that this sort of technologies will improve significantly over the next two decades. However many neuroethical issues and challenges have already been identified with such neuroscience technology but a hypothetical scenario where there is a high demand in 20 years for non-invasive brain augmentation devices that can improve attention, memory, learning, mood, or inter-person communication is considered. It is possible that manufacturer, supplier, or user entities will independently adopt anticipatory steps to manage such dangers. To build ethically directed neurotechnologies that advance humanity to new heights in the near future, we propose acceptable ethical frameworks for standards, government programmes, oversight, and liabilities. The future will demonstrate if we become cyborgs and what we will see when we look back on current neurotechnologies. But the debate about whether and how we should “plug” our brains into technology must start now. We must debate the dangers we are ready to take—and whether there are unexplored roads we do not wish to travel.

## Author Contributions

NJ collected data and drafted the entire manuscript. MS and GA drafted and revised the manuscript. VR, BA, and AA revised the manuscript critically. VS helped in the drafting of figures for the article. All authors contributed to the article and approved the submitted version.

## Funding

This work was funded by the Institutional Fund Projects under grant no. IFPDP-59-22. Therefore, authors gratefully acknowledge technical and financial support from Ministry of Education and Deanship of Scientific Research (DSR), King Abdulaziz University, Jeddah, Saudi Arabia.

## Figures and Tables

**Table:** 

ADHD	Attention Deficit Hyperactivity Disorder
ASD	Autism Spectrum Disorder
BCI	Brain Computer Interface
BMI	Brain Machine Interface
BRAIN	Brain Research through Advancing Innovative Neurotechnologies
Brain/MINDS	Brain Mapping by Integrated Neurotechnologies for Disease Studies
DBS	Deep Brain Stimulation
DIY-tDCS	Do-It-Yourself Transcranial Direct Current Stimulation
DLPFC	Dorsolateral Prefrontal Cortex
ECoG	Electrocorticography
ECT	Electroconvulsive Therapy
EEG	Electroencephalogram
ERP	Event-Related Potential
FDA	Food and Drug Administration
fMRI	Functional Magnetic Resonance Imaging
fNIRS	Functional Near-Infrared Spectroscopy
FUS	Focused Ultrasound
HPET	Human Performance Enhancement Technology
IED	Interictal Epileptic Discharges
ITR	Information Transfer Rate
LGS	Lennox	#x02013;Gastaut Syndrome
MDD	Major Depressive Disorder
MEG	Magnetoencephalography
MEP	Motor Evoked Potential
MNS	Median Nerve Stimulation
MRI	Magnetic Resonance Imaging
rTMS	Repetitive Transcranial Magnetic Stimulation
SCP	Slow Cortical Potential
SMA	Supplemental Motor Area
SSVEP	Steady-State Visually Evoked Potentials
tACS	Transcranial Alternating Current Stimulation
tCS	Transcranial Current Stimulation
tDCS	Transcranial Direct Current Stimulation
TMS	Transcranial Magnetic Stimulation
tPCS	Transcranial Pulsed Current Stimulation
tRNS	Transcranial Random Noise Stimulation
TS	Tourette’s syndrome
tVNS	Transcutaneous Vagus Nerve Stimulation
