# Supplementary figures and images for: Brain augmentation and neuroscience technologies: current applications, challenges, ethics and future prospects
Source: Front Syst Neurosci. 2022 Sep 23;16:1000495. doi: 10.3389/fnsys.2022.1000495 (PMC9538357; doi:10.3389/fnsys.2022.1000495)

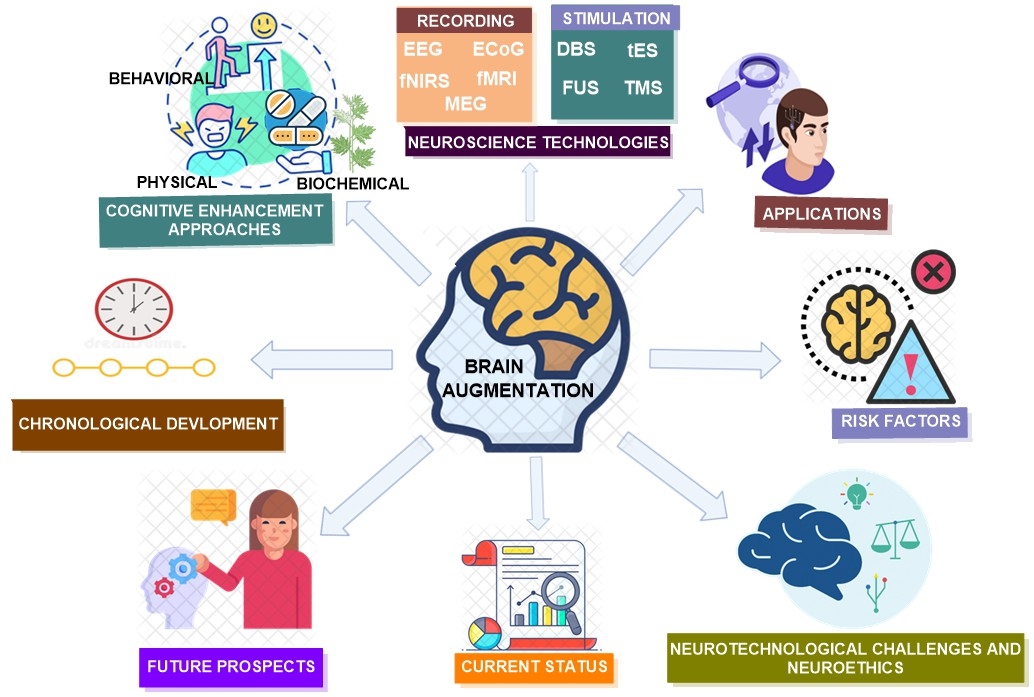

Supplement: Supplementary file 1 [file Image_1.JPEG]
